# Supplementary figures and images for: A deep learning framework for gait-based frailty classification using inertial measurement units
Source: PLoS One. 2026 Feb 24;21(2):e0343402. doi: 10.1371/journal.pone.0343402 (PMC12931800; doi:10.1371/journal.pone.0343402)

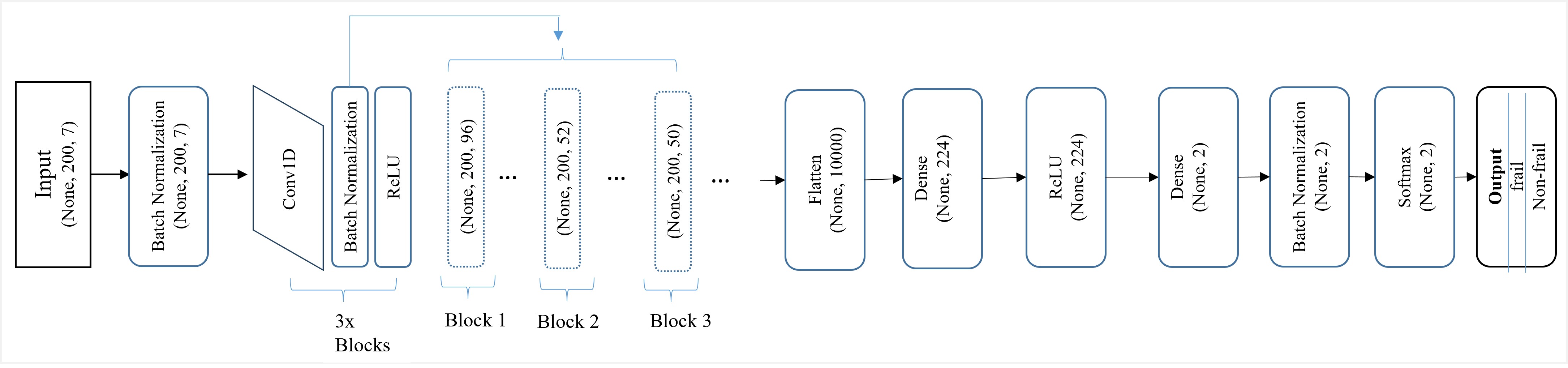

Supplement: S1 Fig — (TIFF) [file pone.0343402.s001.tiff]

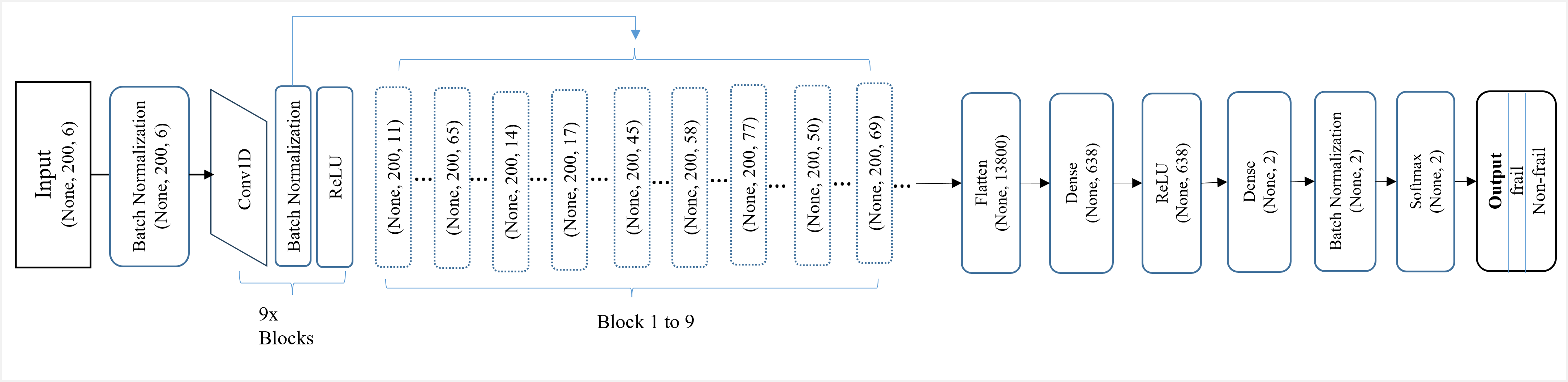

Supplement: S2 Fig — (TIFF) [file pone.0343402.s002.tiff]

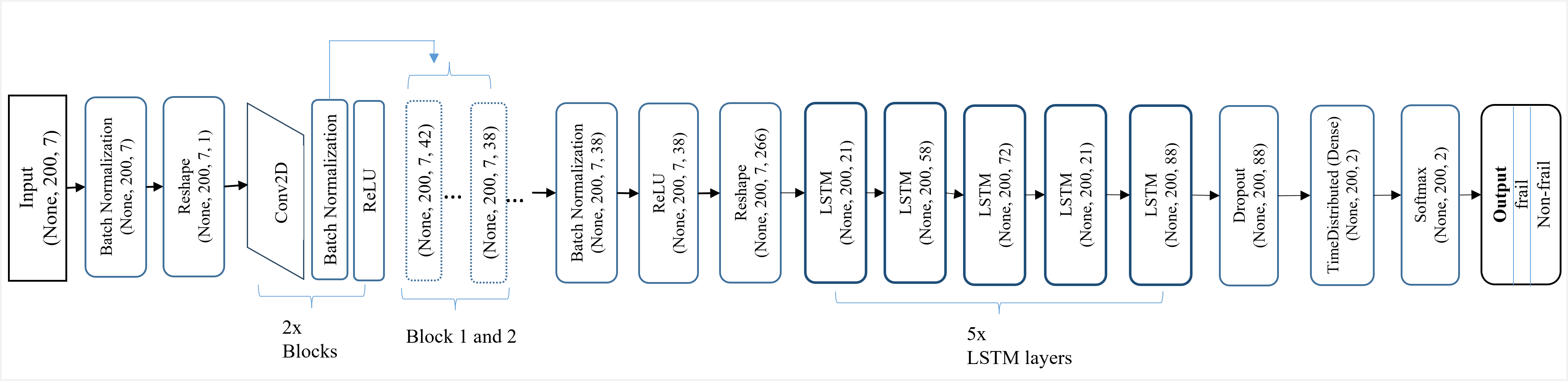

Supplement: S3 Fig — (TIFF) [file pone.0343402.s003.tiff]

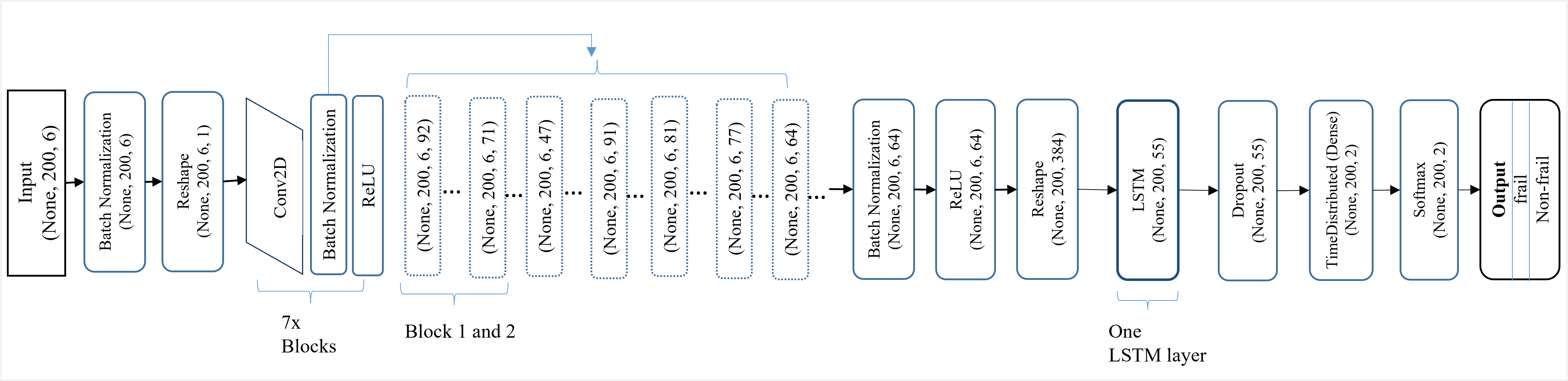

Supplement: S4 Fig — (TIFF) [file pone.0343402.s004.tiff]

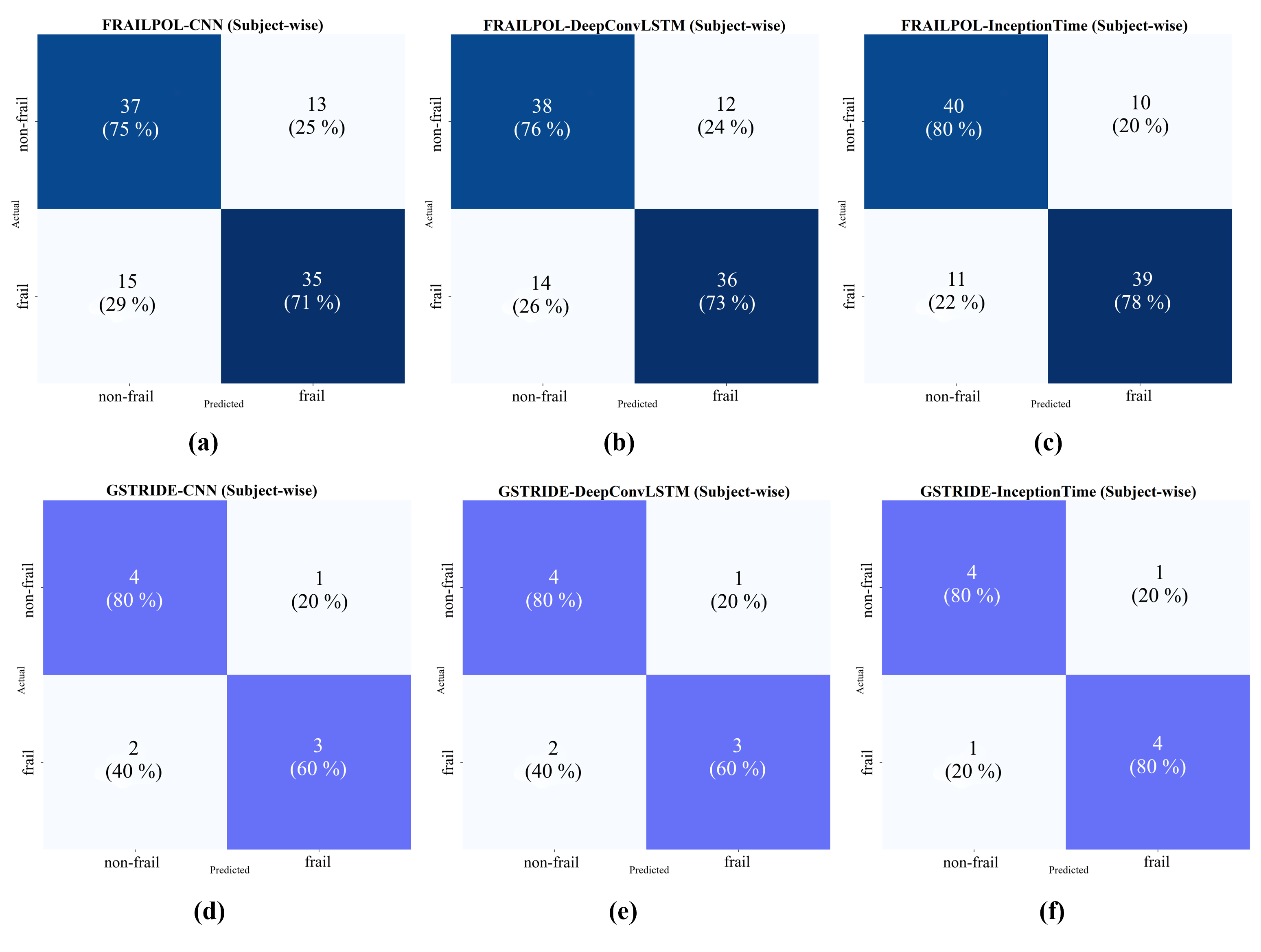

Supplement: S7 Fig — (TIFF) [file pone.0343402.s007.tiff]

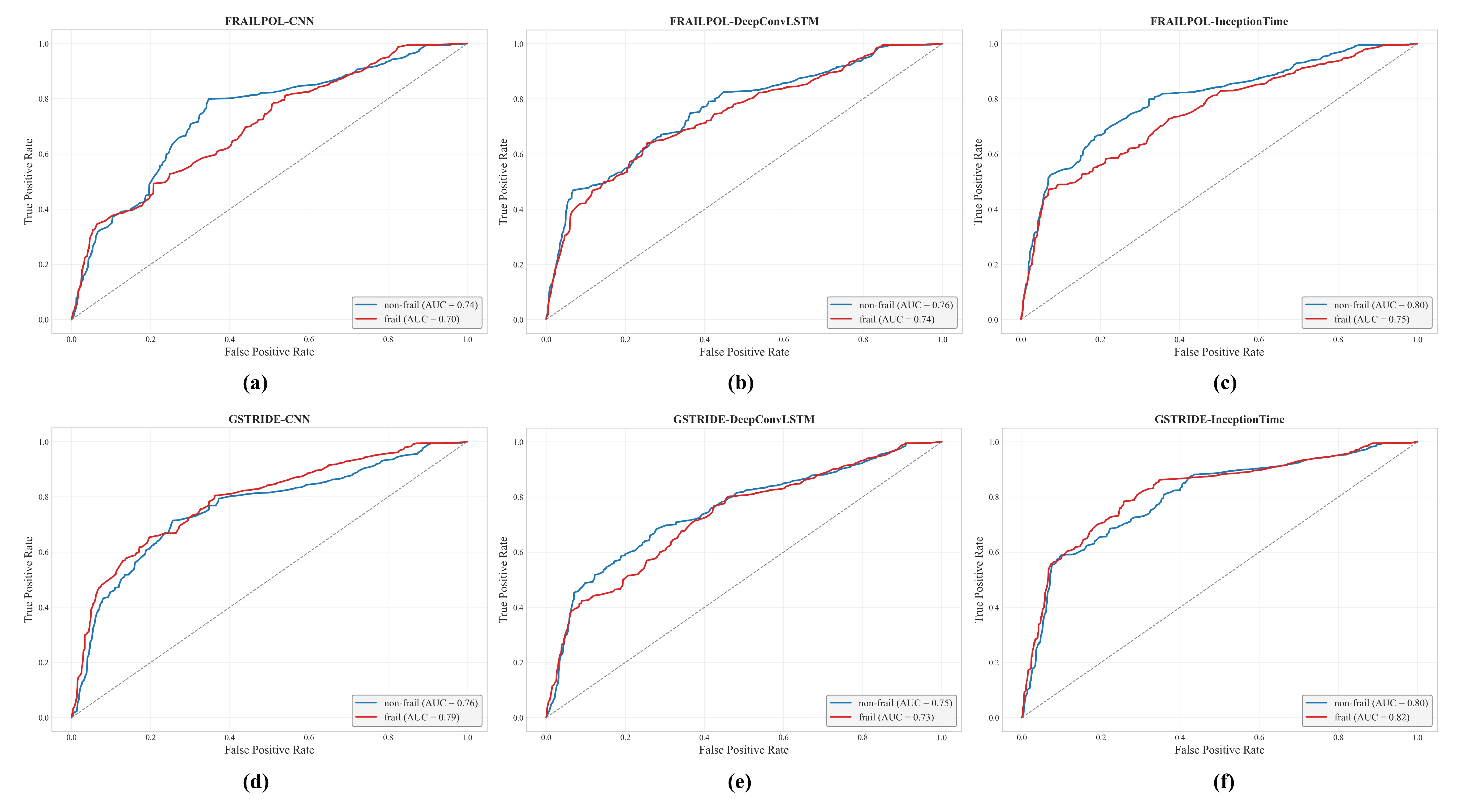

Supplement: S8 Fig — (TIFF) [file pone.0343402.s008.tiff]
