# Supplementary material for: A deep learning framework for gait-based frailty classification using inertial measurement units
Source: PLoS One. 2026 Feb 24;21(2):e0343402. doi: 10.1371/journal.pone.0343402 (PMC12931800; doi:10.1371/journal.pone.0343402)
Supplement: Attachment — Submitted filename: Response to Reviewers.docx [file pone.0343402.s010.docx]

**Dated:** 12-12-2025

**Manuscript ID:** PONE-D-25-50242

**Title:** An Inertial Measurement Unit Based Deep Learning Framework for Personalized Frailty Classification from Gait Signals

**To:** Editor-in-Chief (PLOS ONE)

**Re:** Response to reviewers

Dear Editor,

Thank you for allowing the resubmission of our manuscript, with an opportunity to address the reviewers’ comments. We appreciate the constructive feedback and have carefully revised the manuscript to address all points raised. The reviewers’ suggestions have substantially strengthened the methodology, analysis, and clarity of our work.

Attached below is our detailed point-by-point response to each comment, outlining the specific changes implemented in the revised manuscript. All major revisions have been highlighted in the revised manuscript file for your convenience.

We believe the manuscript has been significantly improved and hope it now meets the journal’s standards for publication.

Sincerely,

Arslan Amjad,

Faculty of Automatic Control, Electronics and Computer Science,

Silesian University of Science and Technology,

Gliwice, Poland

Email: [aabbasi@polsl.pl](mailto:aabbasi@polsl.pl)

**Reviewer#1, Criticism # 1:**

**Data availability:**

- **The study states that the data are ‘fully available,’ but clarifies that the FRAILPOL dataset can only be obtained ‘upon reasonable request.’ This does not guarantee the repeatability of the results.**
- **I recommend making the FRAILPOL dataset public, at least in anonymized form, in a recognized repository (e.g. Zenodo, Figshare, OSF or PhysioNet), including the processed IMU files and participant metadata (age, sex, FI, sensor type, etc.).**
- **If there are ethical or legal restrictions, the authors should clearly justify these limitations and provide a transparent mechanism for access. I am concerned about the independence between the training and testing data. It appears that normalization was applied to both sets in the ML approach. In the DL approach, random sampling was used, with overlapping segments as input data.**

**Author response:** We are thankful to the reviewer for your concern. We have made the FRAILPOL dataset publicly available on the *figshare* repository, as suggested. The dataset can be accessed at the figshare repository (link: <https://doi.org/10.6084/m9.figshare.c.7874411>). This repository contains the processed IMU files and participant metadata for all included participants. The documentation included, *gait_parameters_description.txt* and *raw_imu_signals_description.txt* provided alongside the data for clarity. The FRAILPOL dataset reflects 668 participants. Initially the total participants were 771, but 103 participants were excluded from the study due to either technical error of losing all their sensor files or having incomplete sensor recordings in one file. We have updated the “Data Availability Statement” accordingly to ensure transparent and direct access.

**Author action:** Please refer to subsection 3.1.2 (FRAILPOL Dataset) to the revised version of the manuscript.

**Reviewer#1, Criticism # 2:**

**Section 3. Methodology**

**The description of the methodological framework is too general and should be developed more precisely to ensure the transparency and reproducibility of the study. We recommend:**

**Section 3.3 Data Pre-Processing**

- **Specify what type of filter or noise reduction method was applied to the IMU signals during preprocessing, noting the main parameters (order, cutoff frequency, etc.).**
- **Explain more clearly the order in which time segmentation using the sliding window technique and participant-centric data partitioning are applied, indicating precisely how both stages are integrated into the workflow.**
- **Provide the specific time parameters for the windows (e.g., how many seconds a 200-sample window represents and the justification for the stride of 50), also explaining whether other configurations were evaluated. There is a lack of information on the number of samples per class (statistical significance tests)**

**Author response****:** We are grateful to the reviewer for this comment. Section 3.3 now specifies that a fourth-order, zero-phase Butterworth low-pass filter was applied with cutoff frequencies of 15 Hz for accelerometer and 10 Hz for gyroscope data (lines 223-225).

The participant-centric data partitioning is detailed in section 3.4 (Data Partitioning) (lines 245–260), clarifying that sliding window technique was performed first, followed by participant-based data split within each subset to prevent data leakage.

Regarding temporal parameters, a 200-sample window corresponds to 2.0 s, and a stride of 50 equals a 0.5 s step (indicated in lines 228–233) (section 3.3). These parameters were selected as optimal through a comprehensive evaluation of multiple window sizes and stride lengths.

**Author action:** Please refer to Subsections (3.3) and (3.4) of the revised version of the manuscript.

**Reviewer#1, Criticism # 3:**

**Section 3.4 Data Partitioning**

**The manuscript indicates that 10 of the 163 participants in the GSTRIDE set (and 100 of the 682 in FRAILPOL) were selected to form the test set. However, it does not explain how these subjects were chosen or whether the procedure was repeated multiple times. If the selection were performed only once, the results could depend on that particular partition, compromising the statistical robustness and external validity of the model. It is recommended to describe in detail the selection criterion (random or stratified by class) and, preferably, apply per-participant cross-validation (e.g., k-fold) to obtain more stable and representative performance metrics. Reporting the mean and standard deviation of the metrics across different partitions would significantly strengthen the credibility of the results.**

- **Detail how the participants who make up the training, validation, and test sets were selected, indicating whether the assignment was random or stratified (by age, gender, or frailty level) and whether a balance between classes (frail/non-frail) was maintained in each set.**
- **Specify whether cross-validation or repetition of the process was applied to assess the stability and robustness of the results.**

**Author response:** We thank the reviewer for this critical point. The test sets for both GSTRIDE and FRAILPOL were constructed using a stratified random sampling approach based on participant ID. This ensured that the class distribution (frail or non-frail) in the test set was representative of the entire dataset. To mitigate the risk of results being dependent on a single random partition and to assess stability, the model training and evaluation process was repeated three times with different random seeds for the data split. The performance metrics reported in the results represent the average of these three independent runs.

While we acknowledge that a k-fold cross-validation would provide a more robust estimate of model performance, it was computationally expensive given the large number of participants and the high-dimensional nature of the windowed time-series data. The repeated hold-out validation method was therefore employed as a practical and rigorous alternative to demonstrate model consistency. This explanation has been added to section 3.4 (Data Partitioning) (lines 255-260).

**Author action:** Please refer to Subsection (3.4) (lines 255-260) of the revised version of the manuscript.

**Reviewer#1, Criticism # 4:**

**Section 3.5 Deep Learning Methods and Implementation**

**Although the three architectures used (CNN, DeepConvLSTM, and InceptionTime) are adequately presented, the technical description is primarily descriptive and lacks justification for the design and optimization decisions. It would be advisable to explain why these architectures were selected, how the hyperparameters were tuned (whether through systematic or automatic search), and whether training sessions were repeated with different seeds to ensure stable results.**

**Author response:** We acknowledge the reviewer's valid concern regarding architectural justification and optimization. To address the inherent challenge in selecting and tuning deep learning models, we employed McFly library, an automated machine learning library specifically designed for time-series classification. As referenced in Section 3.5 (Deep Learning Methods and Implementation), the framework systematically explores the hyperparameter space for each architecture (i.e., CNN, DeepConvLSTM, InceptionTime) to identify optimal configurations. For each model type, two architectures were generated and evaluated. The final model was selected based on objective performance criteria, specifically the lowest training and validation loss coupled with the highest validation accuracy. This automated, data-driven approach mitigates selection bias and ensures a reproducible and optimized model development process.

**Author action:** Please refer to Subsection (3.5) of the revised version of the manuscript.

**Reviewer#1, Criticism # 5:**

**Section 4. Results**

**The results section adequately presents the basic performance metrics (precision, recall, F1 score, and accuracy) along with loss curves and confusion matrices. However, there are aspects that need to be improved:**

- **The evaluation metrics are reported as single values without confidence intervals or standard deviations, which makes it difficult to estimate performance variability.**

**Author response:** We thank the reviewer for this valuable suggestion. We have updated the results table (Table 5) to include performance variability. The evaluation metrics (precision, recall, F1-score, and accuracy) are now reported as the mean ± standard deviation across three independent runs, providing a clear estimate of model performance stability.

**Author action:** Please refer to Table 5 of the revised version of the manuscript.

**Reviewer#1, Criticism # 6:**

**Section 4. Results**

- **Figure 5, using a three-dimensional graph, is difficult to read and does not reflect the variability between models; it would be preferable to replace it with a 2D bar chart or boxplots with error bars that allow a quantitative comparison of the results.**

**Author response:** We agree with the reviewer’s concern. Upon careful consideration, we have decided to remove it. The core comparative analysis between the deep learning models is already comprehensively captured in the updated performance table (Table 5) and the confusion matrices presented in Fig. 5. These elements provide a complete, quantitative, and statistically robust basis for evaluating model performance, making the figure redundant. This revision also streamlines the manuscript, improving its conciseness without sacrificing critical information.

**Author action:** Fig. 5 is removed from the revised version of the manuscript.

**Reviewer#1, Criticism # 7:**

**Section 4. Results**

**Confusion matrices (Figure 6) are useful for visualizing classification balance between classes, but they present a significant conceptual problem:**

- **The matrices are presented as absolute values, which makes it difficult to compare datasets of different sizes and models. It would be advisable to normalize the matrices by rows or columns (as a percentage).**
- **The counts are on the order of tens of thousands of instances, even though the test set consists of only 10 participants in GSTRIDE and 100 in FRAILPOL. This indicates that the metrics were calculated at the time window level rather than at the participant level, implying that multiple non-independent observations from the same individual were treated as separate samples. This approach can artificially inflate accuracy and other metrics, compromising the statistical validity of the results. It is recommended to recalculate the metrics considering the actual experimental unit (the participant), or at least report the results from both perspectives (by window and by subject). Metrics should be calculated per subject (averaging across windows) and then averaged across subjects, not directly over all windows together.**

**Author response:** Thank you for these crucial observations. We have addressed both points. First, we have recalculated and normalized the window-level confusion matrices along with the percentage values as shown in Fig. 5 for clearer comparison. Second, we have evaluated performance at the subject level. The subject-wise confusion matrices, which provide the statistically valid assessment, are now included as Fig. S7 in the supporting information.

**Author action:** Please refer to Fig. 5 and Fig. S7 for window-level and subject-wise confusion matrices, respectively. Also refer to lines 368-372 of the revised version of the manuscript.

**Reviewer#1, Criticism # 8:**

**Section 5. Discussion**

**The discussion interprets the results descriptively, but without quantitative analyses to support the conclusions. The improved performance of InceptionTime is attributed to gait length and the proposed partitioning framework, without formally proving it. Furthermore, the metrics are computed at the window level, which limits claims about generalization. The limitations section is too general and should address the most relevant methodological issues.**

**Author response:** We are grateful for the valuable feedback. We have thoroughly revised the discussion section (Section 5) (lines 372-392). We have incorporated a quantitative analysis using updated window-level and subject-wise confusion matrices (Fig. 5 and Fig. S7). The window-level confusion matrices provide statistical proof of performance gains. For example, InceptionTime outperformed with a reduced false negative rate for frail windows by 5-6% on the GSTRIDE and FRAILPOL datasets compared to the CNN and DeepConvLSTM models. The subject-wise confusion matrices (Fig. S7) further validate the performance of the proposed framework.

**Author action:** Please refer to Section 5 (Discussion) of the revised version of the manuscript.

**Reviewer#1, Criticism # 9:**

**Section 6. Conclusion**

**The conclusion summarizes the study's objectives well, but the claims about the model's efficacy and generalizability are too strong considering the available evidence. It is recommended that these statements be expressed with greater caution and that the need for further validation at the participant level and in broader populations be emphasized.**

**Author response:** Taking into account the valuable feedback of the reviwer, we have revised the conclusion to moderate claims regarding model efficacy and generalizability. As indicated in lines 445-448, the text now explicitly acknowledges the preliminary nature of the findings and emphasizes the necessity for future validation with larger, more diverse datasets. Furthermore, multimodal data can also improve frailty assessment in the real-world environment.

**Author action:** Please refer to Section 6 (Conclusion) (lines 455-458) of the revised version of the manuscript.

**Reviewer#2 Criticism # 1: Current versions include numerous instances of awkward phrasing, grammatical errors, and unclear sentences. Some examples are provided here: “The CNN architecture [36] utilized in this study extracted the temporal features…”; Use of “sofmax” in CNN output layer description; “frailty classification from Gait Signals” → “gait signals” should be lowercase. A comprehensive language revision is strongly recommended.**

**Author response:** We thank the reviewer for their feedback. We have conducted a comprehensive language revision of the manuscript to correct grammatical errors, improve sentence clarity, and ensure consistent formatting. All noted issues, including the examples provided, have been addressed.

**Author action:** Please refer to the revised version of the manuscript.

**Reviewer#2 Criticism # 2:** **While two datasets were used in this study, one of them is not publicly available. The authors are encouraged to use more public datasets to validate the generability of the proposed data partitioning method. In addition, a summary table or schematic diagram for each model architecture used in this study will be beneficial for model architecture clarification.**

**Author response:** We are grateful to the reviewer for this suggestion. The primary aims of this study were to evaluate the FRAILPOL dataset against the largest public gait benchmark (GSTRIDE) and to develop a model robust to sensor count and placement variability. While we agree that validation with additional public datasets would strengthen generalizability. Future work should extend beyond gait data by incorporating multimodal data. For example, environmental and physiological factors would make frailty assessment more comprehensive. It would also make it more accurate. As recommended, detailed schematics of each model architecture have been added to the supporting information (figures S1, S2 and S3, S4, S5 and S6) for clarity.

**Author action:** Please refer figures (S1, S2 and S3, S4, S5 and S6) to the revised version of the manuscript.

**Reviewer#2 Criticism # 3: The study did not explore which features or sensor placements contributed most to the classification, further ablation studies are strongly recommended to include.**

**Author response:** We thank the reviewer for this valuable suggestion. The current study was designed to evaluate the feasibility of deep learning models as an initial, end-to-end learning for frailty classification across different IMU sensor mounting positions. By employing a feature-agnostic approach with raw data, we aimed to assess overall model performance without considering spatiotemporal patterns. While an ablation study on sensor placement would be insightful. Our primary comparison between the multi-sensor FRAILPOL setup and the single-foot GSTRIDE dataset serves as end-end learning. It demonstrates that robust classification is possible even with significant variation in sensor count and position. For future work, we agree that a formal ablation study on the contribution of individual sensor placements within the FRAILPOL setup is a logical and necessary step to identify optimal minimal configurations for clinical application.

**Author action:** Please refer to the revised version of the manuscript.

**Reviewer#2 Criticism # 4: Please address or discuss the below limitations in the manuscript:**

- **Limited Diversity in Demographics and Sensor Placement from datasets.**
- **Consider elaborating on how real-time implementation might be approached, given the focus of this manuscript is on clinical applicability. Ideally, computational complexity should be included and evaluated in this study.**
- **The manuscript does not clearly discuss how the proposed method can address the imbalanced dataset issue that might affect model learning performance.**

**Author response:** Taking into account the valuable feedback of the reviwer, we acknowledge the limitations of the current gait datasets (FRAILPOL and GSTRIDE) in demographic and sensor diversity. As mentioned in section 5.1 (Limitations and future work), future research should extend beyond gait data by incorporating multimodal data, including environmental factors and physiological markers, to improve the robustness and comprehensiveness of frailty assessment.

For the second concern, the primary focus of this study was the development and validation of a robust classification methodology. Implementing a real-time system remains a clear objective for future work (as mentioned in section 5.1). We also aim to continue research by integrating more diverse, multimodal datasets and optimizing deep learning models to further improve frailty assessment.

Lastly, class imbalance was addressed through participant-level stratified sampling in data partitioning and by the evaluation of class-specific metrics (precision, recall, F1-score) (section 3.4 and section 4). This approach reduces bias from class prevalence and ensures a fair evaluation of performance for both frail and non-frail individuals.

**Author action:** Please refer to Subsection 5.1 (Limitations and future work), Subsection 3.4 (Data partitioning), and Section 4 (Results) of the revised version of the manuscript.

**Reviewer#3 Criticism # 1: Suggest removing Fig 4, training errors are not very informative for understanding the performance of ML algorithms.**

**Author response:** We appreciate the reviewer's suggestion. While we agree that test set performance is the ultimate metric. We respectfully believe the training and validation loss plots (Fig. 4) provide critical insights for understanding model behavior that are not captured by final metrics alone. These plots are essential for demonstrating that the models were not overfitting (indicated by converging training and validation curves) and that the learning process was stable and converged properly. This transparency is a key component of reproducible deep learning research, allowing readers to assess the quality of the training process that led to the reported results. Therefore, we propose to retain the figure to provide a complete view of model development.

**Reviewer#3 Criticism # 2: Table 4. I am surprised to see there is very little difference across the four types of measures for all datasets and methods compared. Please explain why this is the case. Also, because the sample size of FRAILPOL is much bigger than that of GSTRIDE, I am surprised to see the performance is actually worse. Please explain why this is the case.**

**Author response:** We appreciate the reviewer's observation. We believe the intended reference is Table 5, which contains the comparative performance metrics. The convergence of these four metrics is a direct indicator that our classifiers are performing consistently across both classes. When precision and recall for a binary classifier are similar, the F1-score will naturally align with them. Furthermore, when the model makes a roughly equal number of false positives and false negatives, the overall accuracy also converges toward these values.

Additionally, FRAILPOL has more participants, but GSTRIDE contains substantially more data samples (windows) per participant due to its longer recording protocols. The larger FRAILPOL dataset exhibits slightly lower performance than GSTRIDE not only because of its size, but also because of its substantially higher heterogeneity. The FRAILPOL dataset uses five sensors on various body locations (2x wrists, 2x ankles, and back of sacrum). This introduces a higher-dimensional, more complex, and potentially noisier feature space compared to the single, standardized foot sensor in GSTRIDE.

For a comprehensive analysis, we evaluated performance at both the window level and the subject level. We recalculated and normalized the window-level confusion matrices along with the percentage values as shown in Fig. 5 for clearer comparison. Second, we have evaluated performance at the subject level. The subject-wise confusion matrices, which provide the statistically valid assessment, are now included as Fig. S7 in the supporting information.

**Reviewer#3 Criticism # 3: Fig 5. Why only compare accuracy? How about other measures?**

**Author response:** We are thankful to the reviewer for your concern. The primary metrics, including precision, recall, F1-score, and accuracy for each model and dataset, are fully reported and compared in Table 5. The table provides the complete quantitative analysis that complements the visual summary in the figure. Whereas Fig. 5 presents the normalized window-level confusion matrices. This allows direct visual comparison of per-class recall and precision.

**Author action:** Please refer to Table 5 and Fig. 5 of the revised manuscript.

**Reviewer#3 Criticism # 4: Fig 6. It is fine to use confusion matrix, but a threshold is needed to construct such a matrix. But no information is provided on the threshold and why choose that threshold.**

**Author response:** We thank the reviewer for raising this point. In our study, all deep learning models (CNN, DeepConvLSTM, and InceptionTime) were configured for direct categorical classification. The final layer uses ‘softmax’ activation, and predictions are made by selecting the class with the maximum probability (argmax). This standard approach does not involve an adjustable decision threshold. Therefore, the confusion matrices in Fig. 5 are constructed directly from these argmax predictions.

**Author action:** Please refer to Subsections (3.5.1), (3.5.2) and (3.5.3) of the revised manuscript.

**Reviewer#3 Criticism # 4: I suggest authors to draw ROC curves to compare performance visually.**

**Author response:** We appreciate the reviewer’s suggestion. However, ROC analysis is not well-suited to our study for methodological reasons. Although the test set was constructed using stratified sampling, the training data remains imbalanced. ROC curves are known to produce overly optimistic results under class imbalance because the false-positive rate is dominated by the majority class.
