# Supplementary material for: A deep learning framework for gait-based frailty classification using inertial measurement units
Source: PLoS One. 2026 Feb 24;21(2):e0343402. doi: 10.1371/journal.pone.0343402 (PMC12931800; doi:10.1371/journal.pone.0343402)
Supplement: Attachment — Submitted filename: Response to Reviewers R2.docx [file pone.0343402.s011.docx]

**Dated:** 17-01-2026

**Manuscript ID:** PONE-D-25-50242

**Title:** An Inertial Measurement Unit Based Deep Learning Framework for Personalized Frailty Classification from Gait Signals

**To:** Editor-in-Chief (PLOS ONE)

**Re:** Response to reviewers

Dear Editor,

Thank you for allowing the resubmission of our manuscript, with an opportunity to address the reviewers’ comments. We appreciate the constructive feedback and have carefully revised the manuscript to address all points raised. The reviewers’ suggestions have substantially strengthened the methodology, analysis, and clarity of our work.

Attached below is our detailed point-by-point response to each comment, outlining the specific changes implemented in the revised manuscript. All major revisions have been highlighted in the revised manuscript file for your convenience. Following the reviewer's suggestion, we have updated the manuscript title to: **"A Deep Learning Framework for Gait-Based Frailty Classification Using Inertial Measurement Units.**"

We believe the manuscript has been significantly improved and hope it now meets the journal’s standards for publication.

Sincerely,

Arslan Amjad,

Faculty of Automatic Control, Electronics and Computer Science,

Silesian University of Science and Technology,

Gliwice, Poland

Email: [aabbasi@polsl.pl](mailto:aabbasi@polsl.pl)

**Reviewer#1, Criticism # 1:**

**First, the data partitioning procedure lacks complete transparency and consistency, particularly with regard to the exact number of participants used in the divisions and how stratification was performed at the subject level. This affects reproducibility and needs to be clarified.**

**Author response:** We thank the reviewer for this concern. The total number of participants in non-frail and frail classes of both datasets FRAILPOL and GSTRIDE is now explicitly stated in Subsection 3.2 (Frailty Assessment Criteria) lines 218-221.

To ensure a fair and unbiased evaluation, we employed a participant-level stratified splitting approach. The datasets were partitioned by first creating the test set. Stratified random sampling approach was utilized to select 10 out of 163 participants in the GSTRIDE dataset and 100 out of 682 participants in the FRAILPOL dataset, ensuring proportional class representation. The remaining participants were partitioned in an 80:20 ratio using the stratified *train_test_split* function on the array of unique participant IDs for training and validation sets, respectively (as mentioned in lines 257-262). The stratified method guaranteed that the proportion of frail to non-frail participants in the original dataset was preserved within each split.

**Author action:** Please refer to Subsection 3.2 (Frailty Assessment Criteria) (lines 218-221) and Subsection 3.4 (3.4. Data Partitioning) (lines 257-262) of the revised version of the manuscript.

**Reviewer#1, Criticism # 2:**

**Second, although subject-level evaluation is mentioned, the main results and conclusions are still based primarily on window-level metrics. Given that the clinical objective is the classification of frailty at the individual level, more emphasis should be placed on subject-level performance and, possibly, treated as the primary endpoint.**

**Author response****:** We are grateful to the reviewer for this comment. We agree that the clinical endpoint is ultimately the classification of the individual. The main tables and figures presented results at the window level, which we used to benchmark model architecture and feature performance in line with common practices in IMU-based frailty classification. However, we also reported and discussed the crucial subject-level performance in the revised manuscript. Specifically, the subject-level evaluation was presented in Fig. S7 and Fig. S8.

To address this concern directly and enhance clarity, we have restructured Section 4 (Results) (lines 372-379) to highlight the importance of subject-level metrics. Furthermore, Section 5 (Discussion) has been elaborated to include a dedicated analysis of subject-level performance and its clinical implications (lines 391-398).

A core motivation for our framework was to address the prevalent data leakage problem in studies using sliding windows. Our study explicitly contrasts participant-level evaluation with careful window-level data partitioning framework to demonstrate a generalizable model assessment method.

**Author action:** Please refer to Section 4 (Results) (lines 372-379) and Section 5 (Discussion) (lines 391-398) of the revised version of the manuscript.

**Reviewer#1, Criticism # 3:**

**Thirdly, the use of the term “personalised” remains problematic, as the proposed framework does not include subject-specific modelling, adaptation or personalisation strategies. This constitutes a conceptual exaggeration that should be reconsidered in the title and throughout the manuscript.**

**Author response:** We thank the reviewer for this suggestion. We have changed the manuscript title to: *“A Deep Learning Framework for Gait-Based Frailty Classification Using Inertial Measurement Units”*. Furthermore, we have conducted a careful review of the entire manuscript to ensure the term “personalized” and any related conceptual claims have been removed or rephrased to accurately reflect the study’s generalizable

**Author action:** Please refer to the title of the revised version of the manuscript.

**Reviewer#3 Criticism # 1: The two classes are very balanced. Instead of confusion matrix, I insist that ROC curves to be shown and compared.**

**Author response:** We thank the reviewer for their suggestion. We have added the ROC curves (Fig. S8) for all DL models and included a dedicated discussion section 5 (Discussion) (lines 399-415). The ROC curve illustrates the discriminative performance of DL models across both non-frail and frail classes.

**Author action:** Please refer to Fig. S8 and Section 5 (Discussion) (lines 399-415) of the revised version of the manuscript.
